# Supplementary material for: Assessment of coronary spasms with transluminal attenuation gradient in coronary computed tomography angiography
Source: PLoS One. 2022 Jul 8;17(7):e0271189. doi: 10.1371/journal.pone.0271189 (PMC9269916; doi:10.1371/journal.pone.0271189)
Supplement: S1 Table — (DOCX) [file pone.0271189.s001.docx]

**S1 Table. Diagnostic performance of CCTA for the detection of coronary spasm in per patient and per vessel manner.**

|  | Per patient | Per vessel |
| --- | --- | --- |
| Total no. | 49 | 121 |
| No. of true negatives | 19 | 80 |
| No. of true positives | 24 | 23 |
| No. of false negative | 6 | 15 |
| No. of false positives | 0 | 3 |
| Sensitivity | 80 (61-92) | 60.5 (43-76) |
| Specificity | 100 (82-100) | 96.4 (90-99) |
| Positive Predictive Value | 100 | 88.5 (71-96) |
| Negative Predictive Value | 76 (61-87) | 84.2 (78-89) |
| Accuracy | 87.8 (75-95) | 85.1 (78-91) |

Data are expressed as number or percentage.

Values in parentheses are 95 % confidence intervals
