# Supplementary material for: Assessment of coronary spasms with transluminal attenuation gradient in coronary computed tomography angiography
Source: PLoS One. 2022 Jul 8;17(7):e0271189. doi: 10.1371/journal.pone.0271189 (PMC9269916; doi:10.1371/journal.pone.0271189)
Supplement: Attachment — Submitted filename: Response to reviewers.docx [file pone.0271189.s003.docx]

Dear Editor

First, we would like to thank you for your thoughtful comments on our manuscript entitled, “Assessment of coronary spasms with transluminal attenuation gradient in coronary computed tomography angiography”. The manuscript has been carefully rechecked; moreover, we have extensively made changes as per the reviewers’ recommendations, which have helped us improve the quality of our manuscript. We hope that the revised manuscript is now suitable for publication in *PLOS ONE*. Thank you for your consideration. We look forward to hearing from you.

Point-by-point responses to the comments are provided below.

Response to reviewers

**Reviewer #1:**

**1. Relatively high “false positive VA” (8/57 excluded for fixed stenoses) and “false negative VA” (6/57 excluded for negative CCTA but positive conventional angiogram) in the initial study population. The patients with fixed stenoses are addressed as the second limitation of the study. Please address and account for the false negative patients. These findings suggest that there is significant clinical overlap within the variant chest pain population.**

Answer: Thank you for this thoughtful advice. The six patients showed negative results on CCTA but positive results in the ergonovine provocation test. This could be attributed to individual differences and variations in the timing of CT acquisition. Given the variations in the timing and migratory nature of vasopasms, CCTA studies on coronary spasms have generally discarded such false negative results; accordingly, we exclude them from the results.

Line 517-521; Third, we excluded patients with inconsistent results on CCTA (negative) and the ergonovine provocation test (positive). False-negative results could result from variations in the timing and migratory nature of vasospasms; moreover, they are generally excluded in related studies.

**2. For institutions that perform only CCTA with vasodilator, vasospasm will likely go undiagnosed. Would you recommend adding baseline CT to all variant chest pain CCT protocols in order to help diagnose VA?**

Answer: Thank you for your suggestion. Although our double-acquisition CCTA protocol improved the sensitivity for diagnosing vasospastic angina compared with conventional single-acquisition CCTA protocols (sensitivity in per-patient, 73%; Eur Radiol. 2017;27: 1136–1147), it has several drawbacks in routine clinical practice (radiation dose, contrast media, etc.). Additionally, vasospasms show individual differences at various time during the day, which results in false negative results on CCTA. Moreover, since the spasm provoking test combined with invasive coronary angiography remains necessary for confirming variant angina, our double acquisition CCTA protocol cannot be used an independent diagnostic tool yet.

Line 501-509; In our previous study (9), we examined the feasibility of the double acquisition CCTA protocol, which showed a relatively higher sensitivity for diagnosing VA compared with previous conventional single acquisition CCTA protocols. Moreover, according to present study, we found difference of TAG values of coronary spasms depend on the morphological subtype which may improving the detection of diffuse type spasm. However, due to numerous limitations, including the difficulty of performing CCTA twice for the same patient, variations in the time of vasospasm, increased radiation exposure, increased amount of contrast material used, and the requirement of CAG for confirmatory diagnosis of VA, further research is needed to determine the clinical use of the double acquisition CCTA protocol.

**3. Because hypertension and smoking are so significantly correlated with positive vasospasm patients, is TAG more useful in that population? Please comment on this in the discussion.**

Answer: Thank you for your suggestion. Male sex, smoking, and hypertension are established clinical risk factors for coronary spams, which is consistent with our findings. Given our small sample size and the limited number of patients with those comorbidities (hypertension 11/57, smoking 21/43), we could not analyze the actual correlations between the patients’ characteristics and TAG values. Moreover, we observed that the TAG value is significantly dependent on the type of coronary spasm. Therefore, we cannot guarantee the utility of additional TAG analysis in the detection of coronary spasms on CCTA.

**4. Nondiagnostic coronary segments were manually removed from the analysis (i.e, due to motion or blooming artifact) Can you include details about how many times was this necessary? Were there any entirely nondiagnostic CCTA exams?**

Answer: Based on previous reports regarding TAG measurement, segments with motion or blooming artifacts are generally excluded when calculating the TAG values (JACC Cardiovasc Imaging. 2016;9(9):1074–83.; Radiology. 2016;279(1):75–83.); accordingly, we adapted the previously described methodology. Since these segments usually occupy only a small portion of the entire vessel, no vessels were excluded in this step. Therefore, all vessels were available for TAG analysis. We have revised the sentence for improved clarity.

Line 231-232; Datapoints in segments with motion or blooming artifacts from luminal calcium were excluded when calculating TAG values (21).

**5. Who performed the CCTA analysis. Was it one or more than one radiologist? If more than one, were there are any discrepant results and how were those adjudicated.**

Answer: Thank you for this thoughtful comment. In our study, two radiologists (EJ Kang, HS Yong) independently performed CCTA analysis, with discrepancies being reconciled through consensus. The weighted kappa for agreement between radiologist for decisions regarding coronary spasms were 0.781 (95% confidence interval (CI): 0.601 to 0.960) per patient and 0.759 (95% CI: 0.618 to 0.901) per vessel.

Line 218-220; All CCTA images were independently reviewed by two radiologists (E.J.K and H.S.Y) who were blinded to the patients’ clinical information, and discrepancies in results were resolved through consensus.

Line 257-258; Interobserver agreement for decisions of coronary spasm was assessed using a kappa test.

Line 303-306; The weighted kappa for agreement between the radiologists regarding decisions of coronary spasm were 0.781 (95% confidence interval (CI): 0.601 to 0.960) per patient and 0.759 (95% CI: 0.618 to 0.901) per vessel.

**6. In the discussion, do you mean: We observed that IV nitrate CT shows (?higher?) TAG and higher ProxHU values than baseline CT in all three major coronary arteries. Can you clarify?**

Answer : Thank you for pointing out this unclear point. In our study, the coronary arteries showed an increased TAG value (gentle slope) after vasodilator administration, which could be attributed to their dilatation emphasizing the contrast enhancement effects and increasing the TAG. We have revised this sentence for clarity.

Line 451-452; In our study, IV nitrate CT showed higher TAG (gentler slope) and ProxHU values than baseline CT in all three major coronary arteries.

**Reviewer #2:**

**1. Lines 71-75. I don't understand these sentences. I think the introduction warrants minor expansion to clarify the method of TAG measurement. Also, to explain the influence of vessel diameter on TAG measurement.**

Answer: Thank you for this thoughtful comment. We have modified this section for more clarity.

Line 82-84; However, clinical validation studies have reported conflicting results of the usefulness of using TAG at determining coronary arterial flow (14,15), since TAG may be affected by changes in coronary luminal diameter and collateral vascular enhancement.

**2. Line 140. Were beta blocker or calcium channel blockers used to lower heart rate for CT scans? Was there a difference in the use of these medications in the spasm and non spasm patients? Was the mean heart rate at the time of CT scan the same in both groups (with and without NTG?).**

Answer: Thank you for your thoughtful comment. In our research, we did not administer beta blockers or calcium channel blockers during CT acquisition. There was no significant difference in the heart rate during CT acquisition between the CT protocols.

Line 173-174; We did not administer additional beta-blockers or calcium channel blockers for decreasing the heart rate.

**3. Line 121 and 122. I believe that the character "u" was omitted from the text. The text reads "10-20 g of ergonovine" and "nitroglycerin 100g". Perhaps simply use the abbreviation "ucg" or consult with the editorial staff regarding the use of greek characters.**

Answer: Thank you for your careful observation. We have revised our manuscript accordingly.

Line 152-155; We injected 10–20 μg of ergonovine thrice at 1 min intervals into each coronary artery. Even in the negative cases, oral and intracoronary nitroglycerin (100 μg) and nifedipine (10 mg) were administered before completing the procedure to prevent delayed coronary spasms.

**4. Line 162. I assume that the analysis of TAG was terminated at the distal RCA prior to the bifurcation of the PDA and PLV branch. IF so, this should be clarified in the methods.**

Answer: Thank you for this thoughtful comment. We calculated the TAG of each vessels from the ostium to the distal vessels where the cross-sectional minimal area was < 2 mm^2^, regardless of the branching point, as previously described (JACC Cardiovasc Imaging. 2016 Sep;9(9):1074–83.). We have modified the sentence for more clarity.

Line 229-231; The mean luminal attenuation (HU) was measured at 1 mm intervals, from the ostium to distal levels where the cross-sectional minimal area fell below 2 mm^2^ (12).

**5. Line 185. I believe "distal levels" should be "distal vessels".**

Answer: Thank you for this thoughtful comment. We have revised our manuscript accordingly.

Line 229-231; The mean luminal attenuation (HU) was measured at 1 mm intervals, from the ostium to distal levels where the cross-sectional minimal area fell below 2 mm2 (12).

**6. Line 188. I believe "ostium length" should be changed to "vessel length".**

Answer: Thank you for this thoughtful comment. We have revised our manuscript accordingly.

Line 232-234; TAG was defined as the linear regression coefficient between the intraluminal HU and the distance from the ostium.

**7. Line 266. Is there a minus sign missing from the baseline LAD TAG value?**

Answer: Thank you for this thoughtful comment. We have revised our manuscript accordingly.

Line 345-348; The mean TAG values were significantly lower (steeper slop) for baseline CT than that for IV nitrate CT, especially for LAD and LCX (LAD, -20.92±8.37 vs -16.71±6.90; P<0.001; LCX, -29.56±17.37 vs -22.75±11.97; P=0.001; RCA, -11.50±7.08 vs -9.94±5.67; P=0.068; Table 3).

**8. Was there a difference in coronary dominance between spasm positive and spasm negative patients?**

Answer: Thank you for this comment regarding coronary dominance, which is a very important point in coronary researches. We only included right-side dominant patients since TAG can be influenced by the vessel diameter and length (Br J Radiol. 2018 Jul; 91(1087): 20180043). We have clearly explained this in the revised manuscript.

Line 115-117; Since TAG could be influenced by the vessel diameter and length, we included only right-dominant patients i.e., patients whose posterior descending artery is supplied by the right coronary artery (RCA) (17).

**9. Figure 5. I believe these results are also reported in table 4. I would consider omitting table 4,** **and only use the figure. If you do this, you need to label the results of statistical analysis in the figure.**

Answer: We appreciate your important comment regarding overlapping data. We decided to omit table 4, keep figure 5 (in revised manuscript, figure 6), and add additional statistical analysis in the figure (Line 382).

10. Figure 5. Panel B is incorrectly labeled as IV nitrate CT.

Answer: Thank you for this thoughtful comment. We have revised our manuscript accordingly.

.

11. Figure 5. Panel C is incorrectly labeled as baseline CT.

Answer: Thank you for this thoughtful comment. We have revised our manuscript accordingly.

12. Line 364, discussion. Please change "epithelial" to "endothelial".

Answer: Thank you for this thoughtful comment. We have revised our manuscript accordingly.

Line 476-480; Akasaka et al. (32) reported that vessels with diffuse spasms had significantly lower coronary flow reserves than those with focal spasms, which suggested that focal spasms are associated with localized endothelial dysfunction of the epicardial coronaries with no significant effect on coronary microvascular function.

**Reviewer #3:**

**1. Subjects for this analysis were enrolled in the NAVIGATOR study but according to the references for that study, only 41 subjects with vasospastic angina were enrolled. The authors need to clarify the discrepancy with the reported 57 enrolled subjects. During what dates were subjects enrolled in the NAVIGATOR study?**

Answer: Thank you for this thoughtful comment. Among the 57 enrolled participants, we excluded 8 patients with significant fixed stenosis and 6 patients who showed contradictory results on the CCTA (negative) and ergonovine provocation test (positive), which implies false negative results. Finally, we included 43 patients. The enrolled patients participated in the NAVIGATOR study from March 2017 to April 2019 rather than the study you mentioned (Eur Radiol. 2017 Mar;27(3):1136–47; Cardiology. 2018;139(1):25–32).

**2. The authors excluded 6 subjects with invasive evidence of vasospasm in whom the CCTA did not reveal spasm (false negative). This is a major source of bias, and these subjects should be included in the analysis.**

Answer: Thank you for this thoughtful comment. Accordingly, we have enhanced our discussion regarding false negative. The six patients showed negative results on CCTA but positive results on the ergonovine provocation test, which could be attributed to individual differences and variations in the timing of CT acquisition. Given the variations in the timing and migratory nature of vasopasms, CCTA studies on coronary spasms have generally discarded such false negative results; accordingly, we exclude them from the results.

Line 517-521; Third, we excluded patients with inconsistent results on CCTA (negative) and the ergonovine provocation test (positive). False-negative results could result from variations in the timing and migratory nature of vasospasms; moreover, they are generally excluded in related studies.

**3. Table 2 is incredibly confusing. I am assuming column 3 represents all vessels without and column 4 all vessels with spasm on CCTA. What one cannot tell from that table is how many vessels without spasm on invasive imaging had spasm on CCTA. If the goal of the table was to depict the proportion of spasm type (focal vs diffuse) in the specific arteries of patients with spasm detected by CCTA, a figure would likely be better. On a related note, what was the correlation spasm detected in the same arteries on invasive provocative testing and were the proportions of spasm type similar?**

Answer: Thank you for this thoughtful comment. No vessels showed spasms on CCTA without spasms on the ergonovine provocation test. We modified table 2 more simply, and have added a diagram showing the proportion of spasm types in the specific arteries (Table 2 and Figure 4). We defined focal and diffuse type on CCTA; moreover, we classified spasm-positive vessels according to the aforementioned types.

**4. Table 3 shows mean TAG and ProxHU results and per vessel differences before and after iv NTG. Subjects with and without spasm on invasive testing are lumped together but would it not be more useful to determine if the change in each parameter with NTG is different between subjects with and without invasive evidence of spasm?**

Answer: Thank you for this thoughtful suggestion regarding the evaluation of TAG and ProxHU values according to the presence of spasms. Accordingly, we have added further results and statistical analyses. Regarding the comparison of the TAG values within the same spasm group [spasm (-) and (+) separately] between the baseline and IV nitrate CT, the spasm (+) group showed a larger difference in the mean TAG value than the spasm (-) group in the LCX and RCA [LCX, 43.5% vs. 19.4%; RCA, 22.8% vs. 9.7%; spasm (+) vs. spasm (-)]. Contrastingly, the LAD showed a smaller change in the TAG value in the spasm (+) group than in the spasm (-) group (14.4% vs. 24.8%). Compared with the LAD, the LCX and RCA showed a higher proportion of the diffuse type than the focal type (diffuse type: 100%, LCX; 80%, RCA; 67%, LAD), which could have contributed to our findings

Line 458-465; Comparison of TAG values in baseline CT between spasm(-) and (+) vessels revealed significant differences in LCX, but not in LAD and RCA. This could be attributed to the relatively hypoplastic nature of LCX, and the small sample size which only included diffuse spasm (+) vessels in the LCX. The spasm (+) group showed a larger mean TAG difference than the spasm (-) group for all coronary arteries except for LAD, which showed a smaller change in the spasm (+) group than in the spasm (-) group. Compared with LAD, LCX and RCA showed a higher proportion of the diffuse type than the focal type (diffuse type: 100%, LCX; 80%, RCA; 67%, LAD), which could have contributed to our findings.

**5. The information presented in Table 4 and Figure 4 are identical and therefore redundant. Furthermore, some of the same data is also presented in the text.**

Answer: We appreciate your important comment regarding overlapping data. We decided to omit table 4, keep figure 5 (in revised manuscript, figure 6), and add additional statistical analyses in the figure.

**6. The manuscript would be greatly improved if the authors correlated TAG (or more specifically the change in TAG with iv NRG) to the gold standard invasive provocative testing. Calculation of sensitivity/specificity and positive/negative predictive value would help determine if measuring TAG (or ProxHU) by CCTA is a useful non-invasive method to diagnose vasospastic angina.**

Answer: Thank you for this important comment. Indeed, we desired to calculate more precise and meaningful results, including sensitivity or specificity. However, our primary goal was determining the relationship between the TAG value and spasm types. There were obstacles impeding more elaborate analysis, including the lack of data volume. Moreover, the TAG value only showed significant differences in the LCX, which further impeded analysis of sensitivity or specificity. Therefore, we focused on presenting differences in TAG values between spasm types, rather than calculating statistically precise values. However, we anticipate further research on the feasibility of TAG compared with conventional CAG in VA diagnosis. We have added an additional comment regarding this limitation.

Line 521-524; Fourth, TAG values showed significant differences only in the LCX. Therefore, we focused on presenting differences in the TAG values between the spasm types, rather than presenting the sensitivity or specificity of the CCTA or TAG values in VA diagnosis.

Minor comments:

**1. The authors need to better clarify what type vasospasm to which they are referring. The reader finds it hard to know if they are talking about spasm detected by invasive provocative testing of by CCTA.**

Answer: Thank you for your comment. We defined the focal- and diffuse-type in CCTA; moreover, we classified spasm­-positive vessels according to the aforementioned types.

Line 185-190; Regarding VA diagnosis, the following were the requirements for a positive finding on CCTA: (a) significant focal stenosis with negative remodeling on baseline CT without definite evidence of plaques in a completely dilated artery on IV nitrate CT (“focal-type”), or (b) diffuse small diameter (<2 mm) of a major coronary artery with a beaded appearance on baseline CT that showed complete dilation on IV nitrate CT (“diffuse-type”) (Figs 2 and 3) (10).

2. There are several grammatical errors that should be remedied.

Answer: The manuscript has been carefully rechecked; also we conducted an English editing service to improve the manuscript.

6. PLOS authors have the option to publish the peer review history of their article (what does this mean?). If published, this will include your full peer review and any attached files.

Answer: We agree to publish the peer review history of the article
