# Supplementary material for: Assessment of coronary spasms with transluminal attenuation gradient in coronary computed tomography angiography
Source: PLoS One. 2022 Jul 8;17(7):e0271189. doi: 10.1371/journal.pone.0271189 (PMC9269916; doi:10.1371/journal.pone.0271189)
Supplement: Attachment — Submitted filename: Response to reviewers.docx [file pone.0271189.s004.docx]

Dear Editor

First, we would like to thank you for your thoughtful comments on our manuscript entitled, “Assessment of coronary spasms with transluminal attenuation gradient in coronary computed tomography angiography”. The manuscript has been carefully rechecked again; moreover, we have made additional changes as per the reviewers’ recommendations, which have helped us improve the quality of our manuscript. We hope that the revised manuscript is now suitable for publication in *PLOS ONE*. Thank you for your consideration. We look forward to hearing from you.

Point-by-point responses to the comments are provided below.

Response to reviewers

**Reviewer #1:**

**Reviewer #3:**

**As mentioned in my previous review, the non-invasive diagnosis of patients with vasospastic angina is a worthy goal. As the authors point out in the Introduction “there have been no studies on TAG in coronary spasm” and therefore a study that adequately explores it utility would be useful. The hypothesis underlying this study is that “TAG for CCTA may allow a higher diagnostic performance for coronary spasm than CCTA alone.” Unfortunately, persistent major flaws in this study preclude confirming or refuting that hypothesis. These include:**

**1.** **The authors excluded 6 subjects with invasive evidence of vasospasm in whom the CCTA did not reveal spasm (false negative). This is a major source of bias, and these subjects continue to be excluded by the authors in this revised manuscript. One cannot accurately assess the diagnostic performance of using CCTA with TAG compared to invasive coronary angiography by excluding these subjects.**

Answer:

Thank you for this valuable comment. In agreement, we have now included the six patients who showed negative results on CCTA but positive results on the ergonovine provocation test in the evaluation of the diagnostic performance of CCTA compared to invasive coronary angiography. The results of the per-patient analysis showed that the sensitivity, specificity, positive predictive value, negative predictive value, and accuracy were 80%, 100%, 100%, 76%, and 87.76%, respectively. We also analyzed that with per vessel level, and added the results with supplement table (Table S1). However, based on our results, there was only partial differences of TAG values for regarding the spasm on diffuse type spasm on LCX. Also we found the differences of TAG values between the spasm subtypes (diffuse vs focal). Thus, we could not applied TAG for the additional diagnostic tool of spasm detection on CCTA. We added this on the discussion session for limitation.

Line 107-110; The diagnostic performance of CCTA for the detection of coronary spasm showed that the sensitivity, specificity, positive predictive value, negative predictive value, and accuracy were 80%, 100%, 100%, 76%, and 87.76%, respectively. Per-vessel analysis results are shown in S1 Table.

Line 448-452; Fourth, the results regarding the TAG differences between spasm(+) and (-) vessels, regardless of vessel and spasm types, could not support an improved VA diagnostic performance with the addition of TAG in the CCTA analysis. We therefore focused on presenting differences in the TAG values between the spasm types.

**2. In the places in text and their rebuttal, the Authors appear to shift the purpose of the manuscript and narrow it to a description of the imaging features of coronary spam on CCTA. Given that the performance of CCTA with TAG in diagnosing coronary vasospasm has not been validated against invasive coronary angiography, an analysis of the type of spasm (focal versus diffuse) in this highly selected population is of limited value in an of itself. Furthermore, if that really is the main purpose of this work, the authors need to compare the spasm on CCTA to that observed on coronary angiography, the goal standard.**

Answer:

Thank you for your comment. As your comment, in the previous manuscript, we shifted the purpose from proving the hypothesis “that using TAG for CCTA may allow a higher diagnostic performance for coronary spasm than CCTA alone”, which was suggested in the introduction, to a description of the imaging features of coronary spam on CCTA. We agree with your comment that our results are insufficient to support the former purpose; therefore, we have deleted the sentence from the manuscript.

In addition to CCTA alone, we attempted to evaluate the usefulness of TAG and TAG difference values in diagnosing VA in CCTA. Unfortunately, both the TAG and TAG differences between any of the groups (i.e. normal vs spasm(+) or diffuse type vs focal type) were not significant, except for the analysis of baseline CT TAG values among normal vs focal vs diffuse type in LAD and RCA (Table 4). Thus, in the revised manuscript we have focused on presenting the differences in TAG values between spasm types.

Line 430-436; Moreover, according to the present study, the differences in TAG values of coronary spasms depend on the morphological subtype, which may improve the detection of diffuse type spasm. However, due to numerous limitations, including the difficulty of performing CCTA twice for the same patient, variations in vasospasm timing, increased radiation exposure, increased amount of contrast material used, and the requirement of CAG for confirmatory diagnosis of VA, further research is needed to determine the clinical use of the double acquisition CCTA protocol.

Line 448-452; Fourth, the results regarding the TAG differences between spasm(+) and (-) vessels, regardless of vessel and spasm types, could not support an improved VA diagnostic performance with the addition of TAG in the CCTA analysis. We therefore focused on presenting differences in the TAG values between the spasm types.

**3. The revised Table 3 snow shows the difference in group mean TAG and ProxHU results. A far more useful metric is the per subject differences that could potentially be able to identify subjects with vasospasm.**

Answer:

Thank you for your comment. Accordingly, we calculated the TAG difference values, the intra-subject difference in TAG value between the baseline CT and IV nitrate CT. The results showed non-significant differences in TAG difference values among normal, focal spasm, and diffuse spasm groups in all three vessels. Among vessel subtypes, LCX, of which all spasm(+) vessels were diffuse type, showed a greater TAG difference for diffuse spasm(+) than for normal type, although this was not statistically significant.

Line 349-357; The intra-subject difference in TAG value between the baseline CT and IV nitrate CT shows relatively larger value in diffuse type spasm vessels than in focal type spasm or negative spasm vessels, but statistically insignificant (diffuse type vs focal type vs normal, 6.20±13.77 vs 2.05±6.13 vs 3.83±7.04; P=0.35; Table 5). Among vessel subtypes, LCX, of which all spasm(+) vessels were diffuse type, showed a greater TAG difference for diffuse spasm(+) than for normal type, although this was not significant (diffuse type vs normal, 15.11±25.68 vs 5.38±7.07; P=0.052; Table 5). The other two vessels, LAD and RCA, did not show significant TAG difference values among normal, focal spasm, and diffuse spasm vessels (LAD, P=0.326; RCA, P=0.433; Table 5).

Line 397-399; Moreover, the intrasubject TAG difference in LCX was greater in the diffuse spasm group than in the normal group. This could be attributed to the relatively hypoplastic nature of LCX, and the small LCX sample size which only included diffuse spasm(+) vessels.

**Reviewer #4:**

**The authors present an interesting study and have addressed the previously raised criticisms in a satisfactory manner. Two points remain:**

**1. I don't see an explicit statement that the data is made available. Please add this.**

Answer: Thank you for your comment. All relevant data are within the manuscript, figures, and its Supporting Information files.

**2. Please have the manuscript reviewed by a native speaker. There are still language issues.**

Answer: Thank you for your comment. The manuscript has now been proofread and corrected by a professional editor.
